# Assessing Knowledge, Attitudes, and Practices (KAP) related to green pharmacy among community and hospital pharmacists in Jordan

**DOI:** 10.1186/s12909-026-08600-5

**Published:** 2026-03-02

**Authors:** Derar H. Abdel-Qader, Khalid Awad Al-Kubaisi, Nadia Al Mazrouei, Rana Ibrahim, Ahmed Alhusban

**Affiliations:** 1 Faculty of Pharmacy and Medical Sciences, University of Petra, Amman, Jordan; 2 College of Pharmacy, Department of Pharmacy Practice and Pharmacotherapeutics, University of Sharjah, P.O. Box 27272, Sharjah, United Arab Emirates

**Keywords:** Green pharmacy, Pharmaceutical waste management, Knowledge, Attitudes, Practices, Environmental stewardship, Pharmacists, Jordan

## Abstract

**Background:**

Pharmaceutical pollution is a growing global concern with serious implications for ecosystems, human health, and water quality. In Jordan, this issue is amplified by weak enforcement of disposal regulations and low public awareness, with studies showing as few as 10% of residents return unused medicines to pharmacies. As trusted medication experts, pharmacists are uniquely positioned to advocate for and implement green pharmacy practices, the integration of environmental stewardship across the pharmaceutical lifecycle. However, the extent to which Jordanian pharmacists are equipped and enabled to fulfill this role has not been systematically assessed.

**Objective:**

This study aimed to provide the first comprehensive assessment of the knowledge, attitudes, and practices (KAP) of both hospital and community pharmacists in Jordan regarding green pharmacy and to identify key predictors of their engagement.

**Methods:**

This cross-sectional survey was conducted over a one-month period among 380 licensed pharmacists practicing in community and hospital settings across Jordan’s three administrative regions. A validated, self-administered questionnaire was used to assess KAP, perceived barriers, and recommendations. The primary outcomes were scores for knowledge, attitudes, and practices. Descriptive statistics were used to summarize KAP levels, and three separate multivariable logistic regression models were developed to identify predictors of Good Knowledge (score ≥ 80%), Positive Attitude (score > 27), and Good Practice (score ≥ 23).

**Results:**

The study identified a significant attitude-practice gap. While pharmacists demonstrated overwhelmingly positive attitudes (mean score 4.4 ± 0.7), this did not translate into consistent practice (mean score 2.9 ± 1.2), particularly for actions requiring systemic support. Multivariable logistic regression analyses revealed that prior training was a consistent and powerful predictor across all domains. Good Knowledge (score ≥ 80%) was most strongly predicted by prior training (aOR = 5.21) and practicing in a chain pharmacy (aOR = 1.60). Highly Positive Attitude (mean score > 4.0) was significantly predicted by younger age (< 35 years; aOR = 1.95) and prior training (aOR = 1.80). Critically, Good Practice (mean score ≥ 3.5) was not predicted by attitude, but by structural and educational factors: prior training (aOR = 3.85), practicing in a chain pharmacy (aOR = 2.01), and a hospital setting (aOR = 1.75).

**Conclusion:**

This study, the first to assess comprehensively KAP on this topic in Jordan, underscored that the primary barriers to green pharmacy were systemic, not a lack of professional will. The findings provided an evidence-based roadmap, highlighting the urgent need for national guidelines, targeted training, and supportive infrastructure to empower pharmacists to translate their positive attitudes into meaningful environmental stewardship.

**Supplementary Information:**

The online version contains supplementary material available at 10.1186/s12909-026-08600-5.

## Introduction

Pharmaceutical pollution is a growing global concern with serious implications for ecosystems, human health, and water quality [3, 16, 18, 23]. This issue is amplified in Jordan, where weak enforcement of disposal regulations and low public awareness have exacerbated the threat of pharmaceutical waste contamination [11, 29]. National studies reveal alarming public habits; for example, over half of households store unused medicines improperly [13], and as few as 10% of residents return them to pharmacies, with most being discarded in household waste [11].

As trusted medication experts, pharmacists are uniquely positioned to mitigate this environmental challenge. Their critical role encompasses promoting safe disposal, reducing waste, advocating for sustainable health practices, and educating the public on how to prevent water and soil contamination [4, 5, 27, 28]. Through direct patient counseling and public engagement, pharmacists can reshape disposal habits and raise awareness of the long-term risks associated with pharmaceutical pollution [26, 33]. However, to fully harness this potential, policymakers require empirical data from real-world pharmacy settings to develop effective, context-specific environmental guidelines [6, 15, 20].

This need for evidence leads directly to the concept of “green pharmacy,” which refers to the integration of environmentally responsible practices across all stages of the pharmaceutical lifecycle, from production and prescribing to disposal [12, 17, 30]. Green pharmacy aims to minimize ecological harm through proper waste management and sustainable supply chains [24, 34]. To evaluate its implementation, scholars often apply the Knowledge, Attitudes, and Practices (KAP) model, a structured framework for assessing professional awareness and identifying barriers to the adoption of sustainable principles [12, 32].

Despite the utility of this model, a significant evidence gap exists. Most international research on the environmental roles of pharmacists originates from highly regulated healthcare systems, which differ substantially from Jordan’s context [14, 22, 35]. Regional studies, such as one from the UAE, suggest that even with high awareness, practice remains poor due to systemic barriers like inadequate infrastructure and policy enforcement [4]. Existing studies in Jordan have focused narrowly on general medication disposal practices [11, 13, 29], without a comprehensive exploration of pharmacists’ broader role in promoting green pharmacy.

Furthermore, the integration of green pharmacy into professional competency frameworks is gaining global momentum. The International Pharmaceutical Federation (FIP) has increasingly emphasized environmental sustainability as a core pillar of modern pharmacy practice [10]. However, in Jordan, undergraduate curricula and Continuing Professional Development (CPD) programs have historically lacked specific modules on pharmaceutical waste management, creating a gap between emerging global standards and local practice [7]. To the best of our knowledge, no prior study has systematically assessed the KAP of both hospital and community pharmacists in Jordan regarding green pharmacy. This gap is a significant challenge, as the absence of localized, profession-specific data can lead to ineffective policies and training. Therefore, this study aimed to provide the first comprehensive evaluation of the KAP of Jordanian pharmacists toward green pharmacy principles. The findings will provide policymakers, educators, and healthcare leaders with actionable insights to inform the development of evidence-based curricula, professional training programs, and national drug return initiatives that are both context-sensitive and impactful, ultimately advancing environmental stewardship within pharmaceutical practice.

## Objectives

This study aimed to provide the first comprehensive assessment of the KAP of hospital and community pharmacists in Jordan regarding green pharmacy principles and environmental sustainability. It further sought to identify key demographic and professional predictors that significantly influence pharmacists’ engagement, with the ultimate goal of generating context-specific, evidence-based recommendations for pharmacy education, national waste management policy, and sustainable pharmaceutical service models in Jordan.

## Methods

### Study design and setting

To achieve the study’s objectives, a quantitative, cross-sectional approach was primarily used. Data collection took place over a one-month period (August 2025) to capture a snapshot of current engagement with green pharmacy principles.

### Ethical considerations

The study was approved by the University of Petra’s Institutional Review Board (IRB, Reference: S/18/8/2025).

### Survey instrument

Data were collected using a structured, self-administered questionnaire specifically developed for this study. Prior to pilot testing, the questionnaire’s content and face validity were established by a multidisciplinary panel consisting of five experienced academic and practicing pharmacists, who reviewed the instrument for clarity, relevance, and comprehensiveness.

The questionnaire was originally developed in English and then translated into Arabic by two independent bilingual pharmacy academics. To ensure linguistic equivalence and cultural adaptation, it was back-translated into English by a third academic blinded to the original version. Discrepancies were resolved through a consensus meeting. Construct validity was established through the multidisciplinary panel review and further supported by the clear factor grouping during pilot testing.

The final instrument ensured triangulation of evidence by integrating items from WHO guidelines, FIP policy documents, and peer-reviewed KAP studies [1–3, 5, 11, 13, 18, 23, 27–29, 36] (See supplementary material). The questionnaire consisted of six sections:

- Demographics and Professional Characteristics: Assessed age, gender, highest academic qualification, practice setting (community vs. hospital), pharmacy type (chain vs. independent), years of experience and prior training in sustainability or waste management.
- Knowledge: This section included multiple-choice and Yes/No/Unsure questions covering pharmaceutical disposal methods, environmental impacts, and eco-friendly packaging. Responses were scored (1 = correct, 0 = incorrect/unsure) to yield a knowledge score. Based on the modified Bloom’s cut-off point, a pharmacist who scored ≥ 80% on the knowledge questions was categorized as having good knowledge. The 80% threshold was selected as it represents a high standard of professional competency, distinguishing pharmacists with a comprehensive and actionable understanding from those with only partial awareness [9, 25].
- Attitudes: Measured pharmacists’ attitudes toward sustainability using a 5-point Likert scale (1 = strongly disagree to 5 = strongly agree). Items addressed professional responsibility, patient education, regulatory needs, and medicine reuse. Accordingly, the total score ranged from 7 to 35, with a higher overall score indicating a more positive attitude. Based on modified Bloom usage in KAP (≥ 75% as positive), we classified positive attitude as ≥ 75% of the maximum domain score (≥ 27). The reason for this cut-off value was to identify participants whose average response was firmly in the agree or strongly agree range, thereby separating those with consistently strong positive convictions from those whose views were merely neutral, ambivalent, or negative. The 75% score was selected based on KAP previous studies (Wahidiyat et al. 2021; [21]).
- Practices: Assessed self-reported practices using a 5-point frequency scale (1 = Never to 5 = Always). Items measured counseling on disposal, segregation of expired stock, collaboration with take-back programs, and continuing education. Accordingly, the total score ranged from 6 to 30, with a higher score indicating more frequent engagement. Following modified Bloom practice in KAP behavior domains, good practice was defined as ≥ 75% of the maximum domain score (≥ 23). The reason for this cut-off value was to identify participants whose average response was firmly in the often or always range, thereby separating pharmacists whose behaviors trend towards consistent, routine implementation from those whose actions are only sometimes, occasional or intermittent. The 75% score was selected based on KAP previous studies ([9], Wahidiyat et al. 2021).
- Barriers and Enablers: Used a select all that apply format for participants to identify perceived barriers (e.g., lack of regulation, training) and enablers (e.g., workshops, government support) influencing the adoption of green pharmacy practices.
- Recommendations: Included three open-ended questions to capture pharmacists’ qualitative views on strategies for improvement, the role of education, and the pharmacist’s role in national waste management systems.

To enhance reliability, the questionnaire underwent pilot testing with a small group of pharmacists (*n* = 15) prior to data collection. The internal consistency (Cronbach’s alpha) for the domains was: Knowledge (α = 0.76), Attitude (α = 0.84), and Practice (α = 0.81), all exceeding the 0.70 threshold for reliability.

### Respondents, recruitment procedures, sample size, and data collection

The target population included all licensed pharmacists actively practicing in patient-facing roles within community and hospital settings across the Hashemite Kingdom of Jordan. The sampling frame was the official registry of the Jordan Pharmacists Association (JPA) and actively practicing in pharmacies. Pharmacists working in non-patient-facing roles (e.g., academia, industry), not actively practicing, as well as pharmacy students and trainees, were excluded from the study.

A proportionate stratified random sampling method was used. The sampling frame was first stratified by practice setting (community and hospital) and then by Jordan’s three administrative regions (North, Central, and South). A computer-generated random number sequence was used to select participants from lists within each stratum, ensuring the sample was representative of the national distribution of pharmacies.

Data collection was conducted by a team of trained research assistants, who were senior pharmacy students briefed on the study protocol, ethical considerations, and principles of professional conduct. Each research assistant was assigned a list of randomly selected pharmacies to visit in person.

Upon arrival at each site, the research assistant first approached the pharmacist-in-charge to explain the purpose of the study and seek permission to proceed. The participant in the study was the licensed pharmacist on duty during the visit. If two or more eligible pharmacists were on duty, the research assistant approached the pharmacist who appeared most readily available to participate, in order to minimize disruption to pharmacy workflow.

Each potential participant was provided with a participant information sheet detailing the study’s objectives, procedures, and a guarantee of anonymity and confidentiality. It was emphasized that participation was entirely voluntary. Upon providing written informed consent, the pharmacist was given the paper-based, self-administered questionnaire to complete at their convenience on-site. The research assistants remained available to clarify any non-clinical questions about the survey instrument and collected the completed questionnaire upon completion to ensure data integrity and maximize the response rate.Using the single proportion formula, which is functionally equivalent to online calculators, such as Raosoft®, we determined the minimum required sample size was 380. This calculation was based on Eq. 1 with a 5% margin of error, a 95% confidence interval, and a conservative 50% response distribution to achieve maximum variance.

1 $$n= \frac{{Z}^{2}\times p(1-p)}{{d}^{2}}$$

To achieve the required sample of 380, a total of 432 pharmacists were approached, resulting in a response rate of 87.9%. Non-participation was primarily attributed to time constraints during busy shifts or rejecting to give consent.

### Statistical analysis

Data were analyzed using SPSS software (Version 26.0). Descriptive statistics (frequencies, percentages, means, and standard deviations [SD]) were used to summarize participant characteristics and KAP responses.

Inferential statistics were used to identify predictors of engagement. To compare mean KAP scores between groups, independent samples t-tests (for two groups) and one-way analyses of variance (ANOVA) with post-hoc tests (for more than two groups) were conducted. The assumption of normality for continuous data was assessed using the Kolmogorov–Smirnov test.

To identify independent predictors of high engagement, three separate multivariable logistic regression models were developed. The binary outcomes were defined as: good knowledge (≥ 80%), positive attitude (≥ 27/35), and good practice (≥ 23/30). For each model, variables with a *p*-value < 0.1 in the univariate analysis were entered into the multivariable model. An assessment for multicollinearity was conducted, and variables with a Variance Inflation Factor (VIF) > 5 were handled appropriately. The final odds ratios were reported with 95% confidence intervals (CIs). A *p*-value of < 0.05 was considered statistically significant for all final models. This two-stage variable selection and Bloom-based dichotomization are closely common analytic practice in previous researches ([9, 25], Wahidiyat et al. 2021). While dichotomizing continuous scales can reduce information, we adopt it here for policy-relevant interpretability, a trade-off that is widely acknowledged in methodological literature [8].

Open-ended responses were analyzed and categorized by two independent investigators based on recurring keywords and concepts from the participant responses. Any discrepancies in coding were resolved through consensus discussion to ensure reliability.

## Results

### Participant demographics and professional characteristics

Of the 380 participating pharmacists, the majority were female (63.4%), with a mean age of 44.5 ± 10.9 years. The sample was nearly evenly split between community (51.3%) and hospital (48.7%) settings. Notably, a significant majority (66.3%) reported receiving no prior training in green pharmacy or pharmaceutical waste management. The demographic and professional characteristics of the participants are detailed in Table 1.

**Table 1:** The demographic and professional characteristics of the participants (*N* = 380)

Characteristic	**Category**	**n (%)**
Age (years)	Mean ± SD (Range)	44.5 ± 10.9 (23–65)
Gender	Female	241 (63.4)
	Male	139 (36.6)
Highest Qualification	BPharm	160 (42.1)
	PharmD	108 (28.4)
	MSc	82 (21.6)
	PhD	30 (7.9)
Practice Setting	Community Pharmacy	195 (51.3)
	Hospital Pharmacy	185 (48.7)
Type of Pharmacy	Chain Pharmacy	184 (48.4)
	Independent Pharmacy	196 (51.6)
Years of Professional Experience	< 5 years	100 (26.3)
	5–10 years	95 (25.0)
	11–20 years	105 (27.6)
	> 20 years	80 (21.1)
Have you received any training in green pharmacy, sustainability, or pharmaceutical waste management?	Yes	128 (33.7)
	No	252 (66.3)

### Knowledge, attitudes and practices

Pharmacists’ knowledge revealed a significant dichotomy: while general awareness of the environmental consequences of pharmaceutical waste was high (92.1%), specific knowledge of Jordan’s regulatory framework was poor. Only 34.2% were aware of any official guidelines, and even those who were showed considerable confusion about the responsible authorities.

Despite this knowledge gap, pharmacists expressed overwhelmingly positive attitudes (mean score 4.4/5) and a strong sense of professional ownership, firmly agreeing that educating patients and minimizing environmental impact were their responsibilities. Crucially, they rejected the idea that this duty belonged to other healthcare professionals or policymakers.

However, these positive attitudes did not translate into consistent action, revealing a significant attitude-practice gap. Practices that could be performed individually, such as counseling patients on disposal (mean 3.9), were common. In stark contrast, practices requiring systemic support, like collaborating with authorized take-back programs (mean 2.1) or participating in environmental campaigns (mean 1.8), were rarely performed, highlighting that the gap is driven by a lack of infrastructure rather than a lack of will.

A comprehensive summary of all knowledge, attitude, and practice findings is detailed in Table 2.

**Table 2:** Pharmacist knowledge, attitude and practice on green pharmacy principles and waste disposal (*N* = 380)

Knowledge Items	Correct Response(s)	n (%) or Mean ± SD
Improper disposal of medicines (e.g., flushing into sewage) may cause	All of the above (water pollution, AMR, soil contamination)	350 (92.1)
Which method is recommended for disposing of expired/unused medicines?	Returning to a pharmacy	340 (89.5)
Eco-friendly packaging in pharmacy refers to	All of the above (reduces waste, biodegradable)	335 (88.2)
Are you aware of any official guidelines in Jordan on pharmaceutical waste management?	Yes	130 (34.2)
	No	155 (40.8)
	Unsure	95 (25.0)
If yes, which body do you believe issues these guidelines? ^*^	Ministry of Health	60 (46.2)
	JPA	25 (19.2)
	Ministry of Environment	10 (7.7)
	I am not sure	35 (26.9)
Attitude Item		
Pharmacists have a professional responsibility to minimize the environmental impact of pharmaceuticals		4.7 ± 0.5
Green pharmacy principles should be integrated into daily pharmacy practice		4.6 ± 0.6
National regulations should mandate environmentally safe disposal of medicines		4.6 ± 0.7
Pharmacists should educate patients about safe disposal of unused or expired medicines		4.8 ± 0.4
Eco-friendly packaging is worth higher procurement costs		3.8 ± 1.2
Medicine reuse programs, if monitored by pharmacists, can be safe and beneficial		4.1 ± 0.9
Addressing environmental impact is more the responsibility of physicians and policymakers than pharmacists		2.1 ± 1.1
Practice Items		
I provide guidance to patients about safe disposal of unused/expired medicines		3.9 ± 1.0
I separate expired/unused medicines from other pharmacy waste		3.5 ± 1.2
My pharmacy collaborates with authorized waste collection or take-back programs		2.1 ± 1.1
My pharmacy uses or promotes eco-friendly or minimal packaging where available		3.1 ± 1.3
I have participated in or organized activities promoting environmental sustainability (e.g., awareness campaigns, recycling)		1.8 ± 0.9
I update myself on green pharmacy topics (through training, workshops, or reading)		3.1 ± 1.4

^*^Percentages for this item are calculated based on the subset of respondents who answered “Yes” to the preceding question (*n* = 130)

### Barriers, enablers and recommendations

Pharmacists’ perceptions strongly indicated that the primary obstacles to green pharmacy are systemic and structural. The most frequently cited barriers were a lack of national guidelines (85.0%), insufficient pharmacist training (75.0%), and limited patient awareness (69.7%). Correspondingly, the most desired enablers were direct solutions to these gaps: the establishment of government regulations (90.0%), access to training workshops (87.9%), and public awareness campaigns (84.7%).

These findings were reinforced by open-ended recommendations, which overwhelmingly called for top-down, systemic changes. Pharmacists advocated for mandatory take-back programs, stronger leadership from professional bodies, and the integration of sustainability into both university curricula (90.0%) and mandatory Continuing Professional Development (85.0%). Ultimately, pharmacists identified their own primary role as front-line patient educators (92.1%), positioning themselves as public health advocates ready to lead awareness campaigns and contribute to policy.

The complete findings for perceived barriers, enablers, and recommendations are detailed in Table 3.

**Table 3:** Perceived barriers and enablers to green pharmacy practice (*N* = 380)

Category	Item	Frequency, n (%)
Barriers^*^	Lack of national guidelines/policies	323 (85.0)
	Limited patient awareness	265 (69.7)
	Lack of pharmacist training	285 (75.0)
	Financial constraints	229 (60.3)
	Lack of institutional support	209 (55.0)
Enablers^*^	Training workshops on green pharmacy	334 (87.9)
	Government regulations/incentives	342 (90.0)
	Public awareness campaigns	322 (84.7)
	Support from professional associations	289 (76.1)
	Collaboration with waste management services	266 (70.0)
Recommendations^**^	1. What strategies could improve green pharmacy practices in Jordan?	
	Government Regulations & National Programs	323 (85.0)
	JPA Leadership & Standard Setting	296 (77.9)
	Public Awareness Campaigns	266 (70.0)
	Collaboration with Stakeholders (Physicians, Waste Services)	247 (65.0)
	2. How should sustainability be integrated into pharmacy education or CPD?	
	Integrate into University Curricula	342 (90.0)
	More Training/Mandatory CPD for Pharmacists	323 (85.0)
	3. What is the pharmacist’s role in national disposal and reuse programs?	
	Patient Education & Counseling (Encourage)	350 (92.1)
	Leading Public Awareness Campaigns	285 (75.0)
	Active Collaboration within the Healthcare System	266 (70.0)
	Advocacy through Professional Associations (JPA)	228 (60.0)

^*^Participants could select more than one option for Barriers and Enablers

^**^Participants could mention multiple category in their responses, so percentages represent the proportion of all 380 participants who mentioned each category

### Predictors of pharmacist engagement in green pharmacy

To identify key demographic and professional predictors of engagement, a series of univariable and multivariable logistic regression analyses were performed for three binary outcomes: Good Knowledge (score ≥ 80%), Positive Attitude (score ≥ 27), and Good Practice (score ≥ 23). For each model, variables with a *p*-value < 0.1 in the univariable analysis were included in the final multivariable model to adjust for potential confounding effects.

The analysis for Good Knowledge initially found three significant predictors: having a PhD (COR = 2.51), practicing in a chain pharmacy (COR = 1.65), and having prior training (COR = 5.45). In the final multivariable model, prior training remained the strongest independent predictor (aOR = 5.21), and pharmacy type also remained significant (aOR = 1.60).

For predicting a Positive Attitude, five variables showed a significant crude association: age < 35 years (COR = 2.10), years of experience (both 5–10 and < 5 years), practicing in a hospital setting (COR = 1.55), and having prior training (COR = 1.89). After adjustment in the final multivariable model, only age < 35 years (aOR = 1.95) and prior training (aOR = 1.80) remained as significant independent predictors.

Regarding the predictors for Good Practice, the univariable analysis identified three significant factors: practice setting (COR = 2.05), pharmacy type (COR = 2.40), and prior training (COR = 4.85). All three remained significant independent predictors in the final adjusted model. The strongest predictor was prior training (aOR = 3.85). Pharmacy type was also highly significant, with pharmacists in chain pharmacies being twice as likely to engage in good practices (aOR = 2.01). Finally, practice setting remained a significant predictor (aOR = 1.75). The complete results of the logistic regression analyses are shown in Table 4.

**Table 4:** Univariate and multivariable logistic regression of predictors for good knowledge, positive attitude and good practice (*N* = 380)

Characteristic	Knowledge		Attitude		Practice	
	**COR (95% CI)**	**aOR (95% CI)**	**COR (95% CI)**	**aOR (95% CI)**	**COR (95% CI)**	**aOR (95% CI)**
Age Group (Ref: > 50 years)						
35–50 years	0.96 (0.58–1.59)	-	1.31 (0.80–2.15)	-	1.95 (0.55–6.90)	-
< 35 years	0.98 (0.58–1.65)	-	**2.10 (1.25–3.53)**	**1.95 (1.08–3.51)**	2.80 (0.85–9.25)	-
Gender (Ref: Male)	1.18 (0.76–1.84)	-	1.25 (0.81–1.93)	-	1.15 (0.45–2.95)	-
Highest Qualification (Ref: BPharm)						
PharmD	1.04 (0.65–1.66)	-	1.02 (0.65–1.60)	-	0.55 (0.15–2.01)	-
MSc	1.28 (0.71–2.31)	-	0.88 (0.50–1.55)	-	1.70 (0.50–5.78)	-
PhD	**2.51 (1.01–6.25)**	1.95 (0.75–5.09)	1.05 (0.45–2.46)	-	0.25 (0.04–1.65)	-
Practice Setting (Ref: Community)	1.15 (0.74–1.79)	-	**1.55 (1.01–2.38)**	1.41 (0.91–2.20)	**2.05 (1.15–3.65)**	1.75 (0.34–3.15)
Pharmacy Type (Ref: Independent)	**1.65 (1.03–2.65)**	**1.60 (1.02–2.51)**	1.19 (0.79–1.79)	-	**2.40 (1.50–3.85)**	2.01 (0.46–4.25)
†Years of experience (Ref:> 20 years)						
11–20 years	0.98 (0.57–1.69)	-	1.15 (0.68–1.95)	-	0.95 (0.45–2.00)	-
5–10 years	1.05 (0.59–1.87)	-	**2.25 (1.29–3.92)**	-	1.10 (0.53–2.28)	-
< 5 years	0.94 (0.53–1.66)	-	**2.50 (1.45–4.31)**	-	1.25 (0.62–2.51)	-
Prior Training (Ref: No)	**5.45 (3.38–8.78)**	**5.21 (3.19–8.51)**	**1.89 (1.21–2.95)**	**1.80 (1.13–2.87)**	**4.85 (2.80–8.40)**	3.85 (0.33–4.21)

An assessment for multicollinearity was conducted prior to the final analysis; all Variance Inflation Factors (VIFs) for the variables included in the multivariable model were < 5; indicating no significant collinearity

Bold represents statistically significant at *p*-values (≤ 0.1 in univariate and ≤ 0.05 in multivariate)

Statistically significant *p*-values in the univariate model were included in the multivariable model

*Abbreviations*: *COR* Crude Odds Ratio, *aOR* Adjusted Odds Ratio

^†^Years of experience was excluded from the multivariable model due to high collinearity with age group

## Discussion

This study provides the first comprehensive assessment of the KAP of Jordanian pharmacists regarding green pharmacy, revealing a significant attitude-practice gap. While pharmacists demonstrated overwhelmingly positive attitudes toward environmental stewardship, these beliefs did not translate into consistent practice. The evidence strongly suggests this disconnect is driven by systemic barriers, primarily a lack of formal training, clear national guidelines, and adequate waste management infrastructure, rather than a deficiency in professional will.

A critical finding was the dichotomy in pharmacists’ knowledge. General awareness of the environmental consequences of pharmaceutical waste was high, yet specific knowledge of the Jordanian regulatory framework was markedly low, with over 40% unaware of official guidelines. This knowledge deficit is particularly concerning given the context of low public awareness of proper disposal in Jordan [27, 28]. This pattern aligns with regional trends, where pharmacists in Kuwait and the UAE also demonstrated limited familiarity with official guidelines despite recognizing environmental risks [1, 19]. This widespread confusion likely stems from fragmented regulatory landscapes where health and environmental policies are developed in silos, preventing clear communication to frontline practitioners [12]. Such ambiguity is a critical barrier, undermining pharmacists’ ability to educate the public and confidently implement policies [35]. Our findings regarding the knowledge deficit in regulatory frameworks (over 40% unaware) highlight a critical need for the JPA to align local competency standards with the FIP Global Competency Framework. Specifically, environmental stewardship should be transitioned from an ‘optional interest’ to a ‘core professional competency’ within mandatory CPD cycles.

In contrast, the uniformly positive attitudes observed represent a significant strength and a potential driver for change. Jordanian pharmacists showed a strong sense of professional ownership, a finding consistent with studies in Kuwait [1] but contrasting sharply with lower positive attitudes reported in the UAE [19], a difference that could be partly attributed to methodological variations or social desirability bias in KAP surveys [22]. Notably, younger and less experienced pharmacists held more positive attitudes, suggesting recent graduates may be more attuned to sustainability issues and represent a receptive cohort for integrating green pharmacy principles into practice [34]. However, as seen in other contexts, translating this positive intention into sustainable practice remains the central challenge^2^.

The attitude-practice discrepancy is a well-documented phenomenon regionally. In Kuwait and the UAE, high environmental awareness and responsibility did not prevent the majority of pharmacists from engaging in improper disposal practices, underscoring that positive attitudes alone are insufficient without enabling systems [1, 19]. Our predictive analysis confirmed this, showing that the strongest predictors of good practice were external, structural factors, prior training, and working in a hospital or chain pharmacy, not individual conviction. Specifically, multivariable logistic regression identified practice setting as a significant independent predictor of good practice. This indicates that pharmacists in hospital settings were significantly more likely to implement green pharmacy practices compared to their counterparts in community pharmacies. This difference is likely due to the more structured protocols and institutional oversight typically found in hospital environments, which are often absent in independent community settings. The significant predictor of prior training aligns with recent findings by Alhamad et al. [5], who noted that Jordanian pharmacists are eager for structured guidance but feel ‘clinically isolated’ from environmental policy. Similarly, the superior practice scores in hospital settings mirror the systematic review by Pitard et al. [31], which emphasizes that institutional infrastructure is the primary determinant of sustainable healthcare delivery, surpassing individual pharmacist intent. This underscores that individual-led interventions have limited success without a functioning collection infrastructure that pharmacists can confidently direct patients [4].

The barriers, enablers, and recommendations identified by participants reinforce these statistical findings and offer a clear roadmap for policy. Pharmacists overwhelmingly called for top-down, systemic solutions, including government regulation, professional association leadership, and mandatory training. These recommendations resonate with research on other systemic challenges in Jordanian pharmacy [2] and echo global calls for systemic infrastructure and coherent policies to support green pharmacy [20, 24]. Essentially, Jordanian pharmacists are advocating for the core tenets of the green pharmacy concept, a system-wide approach encompassing policy, education, and practice [18, 34] and for the type of coordinated national infrastructure that has proven effective in countries like Sweden [32]. Their collective voice provides a context-specific yet globally validated framework for advancing environmental stewardship in Jordan.

While this study’s strengths include its novel, comprehensive scope and mixed-methods design, it has limitations. Its cross-sectional nature precludes causal inferences, and the reliance on self-reported data may be subject to social desirability bias. Furthermore, recruitment relied on pharmacist availability, which could introduce an element of convenience sampling. Nonetheless, the core finding of a systemic attitude-practice gap is highly consistent with international evidence, confirming the global relevance of its conclusions.

## Conclusion

Despite strong positive attitudes toward environmental responsibility, Jordanian pharmacists face a significant attitude-practice gap due to systemic barriers like a lack of guidelines and training. Good practice is driven by structural factors, not personal will. To empower pharmacists to translate their positive attitudes into action, policymakers must create an enabling environment with clear policies, integrated education, and supportive infrastructure.

## Supplementary Information

Supplementary Material 1.

Supplementary Material 2

## Data Availability

Data are available in the supplementary material.
