# Supplementary material for: Assessing Knowledge, Attitudes, and Practices (KAP) related to green pharmacy among community and hospital pharmacists in Jordan
Source: BMC Med Educ. 2026 Mar 2;26:476. doi: 10.1186/s12909-026-08600-5 (PMC13019981; doi:10.1186/s12909-026-08600-5)
Supplement: Supplementary file 1 — Supplementary Material 1. [file 12909_2026_8600_MOESM1_ESM.docx]

***Green Pharmacy KAP Survey for Pharmacists in Jordan***

**Research Question:**
What are the levels of knowledge, attitudes, and practices related to green pharmacy among community and hospital pharmacists in Jordan, and what factors influence them?

**Section A: Demographics and Professional Background**

1. Age (years): ___
2. Gender: ☐ Male ☐ Female ☐ Prefer not to say
3. Highest qualification: ☐ BPharm ☐ PharmD ☐ MSc ☐ PhD ☐ Other: ___
4. Current practice setting: ☐ Community pharmacy ☐ Hospital pharmacy ☐ Other (Academia, Industry, etc.)
5. Type of pharmacy: ☐ Chain pharmacy ☐ Independent pharmacy
6. Years of professional experience: ☐ <5 ☐ 5–10 ☐ 11–20 ☐ >20
7. Have you received any training in green pharmacy, sustainability, or pharmaceutical waste management? ☐ Yes ☐ No

**Section B: Knowledge**

1. Improper disposal of medicines (e.g., flushing into sewage) may cause:
   - ☐ Water pollution
   - ☐ Antimicrobial resistance
   - ☐ Soil contamination
   - ☐ All of the above ✅ *(correct)*
2. Which method is recommended for disposing of expired/unused medicines?
   - ☐ Throwing in household trash
   - ☐ Returning to a pharmacy ✅ *(correct)*
   - ☐ Flushing in toilet
   - ☐ Burning at home
3. Eco-friendly packaging in pharmacy refers to:
   - ☐ Packaging that reduces plastic/aluminum waste
   - ☐ Use of biodegradable or recyclable materials
   - ☐ Smaller unit-dose packs to reduce waste
   - ☐ All of the above ✅ *(correct)*
4. Are you aware of any official guidelines in Jordan on pharmaceutical waste management?
   - ☐ Yes
   - ☐ No
   - ☐ Unsure
5. If Yes, which body do you believe issues these guidelines?
   - □ Ministry of Health
   - □ Jordan Pharmacists Association (JPA)
   - □ Ministry of Environment
   - □ I am not sure

**Section C: Attitudes**

Scale: 1 = Strongly disagree -5 = Strongly agree

1. Pharmacists have a professional responsibility to minimize the environmental impact of pharmaceuticals.
2. Green pharmacy principles should be integrated into daily pharmacy practice.
3. National regulations should mandate environmentally safe disposal of medicines.
4. Pharmacists should educate patients about safe disposal of unused or expired medicines.
5. Eco-friendly packaging is worth higher procurement costs.
6. Medicine reuse programs, if monitored by pharmacists, can be safe and beneficial.
7. Addressing the environmental impact of medicines is more the responsibility of doctors and policymakers than pharmacists.

**Section D: Practices**

Scale: Never – Rarely – Sometimes – Often – Always

1. I provide guidance to patients about safe disposal of unused/expired medicines.
2. I separate expired/unused medicines from other pharmacy waste.
3. My pharmacy collaborates with authorized waste collection or take-back programs.
4. My pharmacy uses or promotes eco-friendly or minimal packaging where available.
5. I have participated in or organized activities promoting environmental sustainability (e.g., awareness campaigns, recycling).
6. I update myself on green pharmacy topics (through training, workshops, or reading).

**Section E: Barriers and Enablers**

**Barriers** (tick all that apply):

- ☐ Lack of national guidelines/policies
- ☐ Limited patient awareness
- ☐ Lack of pharmacist training
- ☐ Financial constraints
- ☐ Lack of institutional support

**Enablers** (tick all that apply):

- ☐ Training workshops on green pharmacy
- ☐ Government regulations/incentives
- ☐ Public awareness campaigns
- ☐ Support from professional associations
- ☐ Collaboration with waste management services

**Section F: Recommendations (Open-Ended)**

1. What strategies could improve green pharmacy practices in Jordan?
2. How should sustainability concepts be integrated into pharmacy education or CPD?
3. What role should pharmacists play in national medicine disposal and reuse programs?
